# The CNCDs and the NTDs: Blurring the Lines Dividing Noncommunicable and Communicable Chronic Diseases

**DOI:** 10.1371/journal.pntd.0000312

**Published:** 2008-10-29

**Authors:** Peter J. Hotez, Abdallah S. Daar

**Affiliations:** 1 Department of Microbiology, Immunology, and Tropical Medicine, The George Washington University and Sabin Vaccine Institute, Washington, D.C., United States of America; 2 Program on Life Sciences, Ethics and Policy, McLaughlin-Rotman Centre for Global Health, University Health Network and University of Toronto, Toronto, Ontario, Canada

World Health Organization statistics show that deaths worldwide from chronic noncommunicable diseases (CNCDs) now exceed those from infectious diseases [1]. The major CNCDs causing the greatest share of deaths and disability worldwide include cardiovascular conditions (mainly heart disease and stroke), some cancers, chronic respiratory conditions, and type 2 diabetes [2]. Together they account for 60% of all deaths worldwide [2]. Approximately 80% of the CNCD deaths occur in low-income and middle-income countries [2]. Most of the factors contributing to such large numbers of deaths, a large proportion of which are premature, are similar to those in developed countries. They include increasing tobacco use, diminished physical activity and lack of exercise, and the consumption of unhealthy foods [2].

CNCDs are defined as diseases or conditions that affect individuals over an extensive period of time and for which there are no known causative agents that are transmitted from one affected individual to another [2]. More than any other type of infection, the neglected tropical diseases (NTDs) most closely resemble the CNCDs. The characteristic feature of the NTDs are their chronic and insidious clinical manifestations, and the resulting long-term disability [3],[4]. An impoverished person suffering from a NTD will typically have the condition for years, sometimes decades, and sometimes their entire life [3],[4]. Indeed, except for the fact that we know their causative agents, the NTDs for the most part meet CNCD-defining criteria. Shown in Table 1 are the major chronic disease syndromes that result from NTDs. Chagas disease is a leading cause of chronic cardiovascular disease in Latin America, often resulting in severe cardiomyopathy [5], while *Loa loa* and other parasitic infections have been identified as possible etiologies of endomyocardial fibrosis in sub-Saharan Africa [6]. Urinary schistosomiasis is a leading cause of bladder cancer in Africa and the Middle East (causing a unique squamous cell carcinoma) [7],[8], while opisthorchiasis and clonorchiasis, both causes of oriental liver fluke infection, cause bile duct carcinoma in Southeast Asia and China [9]. Worldwide, trichuriasis causes more inflammatory bowel disease than either Crohn disease or ulcerative colitis [10], and schistosomiasis is a leading cause of chronic renal disease and failure in Africa and the Middle East [7],[8],[11]. In Asia, paragonomiasis ranks with tuberculosis and lung cancer as a leading cause of hemoptysis [12], and toxocariasis is emerging as an important cause of asthma [13].

**Table 1:** CNCD-Like Syndromes Caused by the NTDs.

Chronic Condition	NTDs as Etiologies	Approximate Number of Cases of Each Infection	Major Geographic Distribution
**Cardiovascular disease**			
Cardiomyopathy	Chagas disease	8–9 million	Latin America
Endomyocardial fibrosis	Loiasis (and other helminthiases)^a^	13 million	Sub-Saharan Africa
**Cancer**			
Bladder cancer; squamous cell carcinoma	Urinary schistosomiasis (*S. haematobium* infection)	119 million	Africa
Bile duct carcinoma	Opisthorchiasis and clonorchiasis	6–44 million	Southeast Asia and China
**Gastrointestinal and liver disease**			
Inflammatory bowel disease	Trichuriasis	604 million	Developing countries
Megacolon and megaesophagus	Chagas disease	8–9 million	Latin America
Intestinal and liver fibrosis	Schistosomiasis (*S. mansoni* infection and *S. japonicum* infection)	68 million	Africa, Brazil, and East Asia
Liver cyst	Amebiasis	ND	India, Latin America
Liver cyst	Echinococcosis	ND	Developing countries
**Chronic renal disease**			
Hydronephrosis and renal failure	Urinary schistosomiasis	119 million	Africa
**Blood dyscrasias**			
Anemia	Hookworm infection	576 million	Developing countries
Anemia	Schistosomiasis	207 million	Developing countries
Pancytopenia	Leishmaniasis	12 million	India, Africa, Brazil
**Chronic respiratory conditions**			
Hemoptysis	Paragonimiasis	21 million	East Asia
Asthma	Ascariasis	807 million	Developing countries
Asthma	Toxocariasis	ND	Worldwide

a Still under investigation.

ND, not determined.

Anemia is one of the best documented examples of a chronic condition in which a single NTD, such as hookworm infection, accounts for a significant percentage of the attributable risk [14]–[17], or in which multiple NTD coinfections and polyparasitism make a significant contribution [18]–[23]. Another is cancer—urinary schistosomiasis was shown to account for 28% of the bladder cancer in Bulawayo, Zimbabwe [24], and 0.1% of the world's cancer burden [25], while liver flukes (e.g., *Clonorchis sinensis* and *Opisthorchis* spp.) account for an estimated 0.02% of all cancers [25]. Van der Werf et al. determined that *Schistosoma haematobium* was responsible for 10 million cases of hydronephrosis in sub-Saharan Africa, and *S. mansoni* was associated with 8.5 million cases of hepatomegaly in the region [11]. It has been further estimated that approximately 5.4 million people will develop chronic Chagas heart disease, while 900,000 will develop severe enlargement of the digestive tract (megacolon and megaesophagus) [26].

However, the full extent to which the NTDs listed in Table 1 contribute to the other CNCDs requires active investigation. Among the bottom billion living in the poorest areas of the developing world, the underlying causes of chronic cardiovascular, renal, hepatic, and gastrointestinal disease, as well as cancer, are frequently neglected and unstudied. Because they are so common in low-income and middle-income countries, it is of critical importance to determine how the NTDs contribute significantly to the CNCD burden in such regions. Such an evidence base is critical for informing new policies for tackling chronic disease in developing countries. The new Grand Challenges in CNCDs initiative [2] is an ambitious effort to raise public awareness of these conditions in the developing world, enhance economic, legal, and environmental policies, modify risk factors, mitigate the health impacts of poverty and urbanization, engage the community, and reorient health systems away from treatment towards prevention [2]. Wherever the NTDs geographically overlap with the CNCDs, there is a need to assess the contribution of the former, and to recognize that when it comes to NTDs, the distinction between noncommunicable and communicable diseases can be murky.
